# The extraction of drug-disease correlations based on module distance in incomplete human interactome

**DOI:** 10.1186/s12918-016-0364-2

**Published:** 2016-12-23

**Authors:** Liang Yu, Bingbo Wang, Xiaoke Ma, Lin Gao

**Affiliations:** School of Computer Science and Technology, Xidian University, Xi’an, 710071 People’s Republic of China

**Keywords:** Drug-disease correlations, Module distance, Combined protein network, Incomplete human interactome

## Abstract

**Background:**

Extracting drug-disease correlations is crucial in unveiling disease mechanisms, as well as discovering new indications of available drugs, or drug repositioning. Both the interactome and the knowledge of disease-associated and drug-associated genes remain incomplete.

**Results:**

We present a new method to predict the associations between drugs and diseases. Our method is based on a module distance, which is originally proposed to calculate distances between modules in incomplete human interactome. We first map all the disease genes and drug genes to a combined protein interaction network. Then based on the module distance, we calculate the distances between drug gene sets and disease gene sets, and take the distances as the relationships of drug-disease pairs. We also filter possible false positive drug-disease correlations by *p*-value. Finally, we validate the top-100 drug-disease associations related to six drugs in the predicted results.

**Conclusion:**

The overlapping between our predicted correlations with those reported in Comparative Toxicogenomics Database (CTD) and literatures, and their enriched Kyoto Encyclopedia of Genes and Genomes (KEGG) pathways demonstrate our approach can not only effectively identify new drug indications, but also provide new insight into drug-disease discovery.

## Background

Drug development is expensive, time consuming and has a high risk of failures. On average, it now takes around 14 years [1] and $800 ~ $1000 million to bring a single drug to market [2]. To overcome these problems, more and more researchers have focused on inferring drug-disease relationships by computational approaches, commonly referred to as “Drug Repositioning” or “Drug Repurposing”. Drug repositioning is the application of known drugs and compounds to new indications (i.e., new diseases) [3]. Using drug repositioning, pharmaceutical companies have achieved a number of successes, for example Pfizer's Viagra in erectile dysfunction [4] and Celgene's thalidomide in severe erythema nodosum leprosum [5].

With the dramatic expansion of large-scale genomic, transcriptomic and proteomic data, computational approaches to predict new drug-disease associations have become one of the leading ways. For example, in 2016, Huang et al. [6] developed a novel pipeline of drug repositioning to analyze four lung cancer microarray datasets, enriched biological processes, potential therapeutic drugs and targeted genes for non-small cell lung cancer (NSCLC) treatments. They integrated two approaches: machine learning algorithms and topological parameter-based classification. Zheng et al. [7] proposed a novel weighted ensemble similarity (WES) algorithm to predict the drug-target direct interactions, which provided a potential in silico model for drug repositioning and discovery. Wang et al. [8] developed a new strategy in 2015, which integrated two types of drug repositioning methods. Based on integration of chemical, gene and disease networks, Cheng et al. [9] inferred chemical hazard profiles, identified exposure data gaps, and incorporated genes and disease networks into chemical safety evaluations. With increasing evidence in genetic and molecular biology, we find most diseases reflect the interaction of multiple molecular components [10–13]. Therefore, we should consider the relevant interactions of disease-associated genes in the context of the human interactome [14–17], which point out the therapeutic importance of modules. In 2016, Luo et al. [18] utilized some comprehensive similarity measures and Bi-Random walk (BiRW) to develop a method named MBiRW to identify potential novel indications for a given drug. Yu et al. [19] proposed a method based on known protein complexes to infer drug-disease associations in 2015. PREDICT (PREdicting Drug IndiCaTions) [20] is based on the observation that similar drugs are indicated for similar diseases, and utilizes multiple drug–drug and disease–disease similarity measures for the prediction task.

However, high-throughput methods currently include less than 20% of all potential pairwise protein interactions in the human cell [21–26], which means that we seek to discover drug and disease associations relying on interactome maps that are 80% incomplete. Additionally, the gene lists of diseases and drugs remain incomplete [21–26]. Because of the incompleteness of the interactome and the limited knowledge of disease- and drug-associated genes, it is not clear if the available data have sufficient coverage to map out modules associated with each disease and each drug. Therefore, in order to identify the location of disease modules within the incomplete interactome, Menche et al. [27] presented a new module distance and used the overlap between the modules to predict disease-disease relationships. The module distance can be extended to address other questions at the forefront of network medicine. Furthermore, it discriminates known drug-disease pairs from unknown drug-disease pairs better than most of the existing similarity-based methods, such as the shortest path distance between their targets in the interactome, common targets, chemical similarity, etc. [28]. Hence based on the module distance [27], we propose a new network-based framework to extract drug-disease correlations. First, we map all the disease- and drug-associated genes to a combined protein interaction network. Then based on the module distance [27], we calculate the distances between each pair of drug gene set and disease gene set, and take the distances as the relationships of drug-disease pairs. We also filter possible false positive drug-disease correlations by *p*-value. Finally, we validate the top-100 drug-disease associations related to six drugs in the predicted results. The overlapping between our predicted correlations with those reported in Comparative Toxicogenomics Database (CTD) [29] and literatures, and their enriched KEGG pathways [30, 31] demonstrate our approach can effectively identify new drug indications. Furthermore, it can offer new insight into drug discovery.

## Methods

### Datasets

#### Drug and target data

Drugs and their corresponding targets are downloaded from KEGG database [30, 31] and DrugBank [32]. We combine two datasets and get 3,613 drugs, 1,504 targets, and 11,170 drug-target pairs. Each drug is represented by its KEGG Drug ID and each target is represented by its Entrez gene ID.

#### Disease and gene data

Diseases and their related genes are downloaded from KEGG database. In this study, we focus on cancers, so we get 55 cancer diseases, 2,255 associated genes, and 3,800 disease-gene pairs in all. Diseases are represented by its KEGG Disease IDs and genes are represented by Entrez gene IDs.

#### Human interaction network data

We download a complete and currently feasible interactome from ref. [27], which combines seven different interactions. Their details are shown in the supplementary files of ref. [27]. The combined network is scale-free, which includes 13,460 human proteins and 141,296 unique pairwise binary interactions. It is well connected and has small mean clustering coefficient and short shortest path [27]. Its topological properties are shown in Table 1.

**Table 1:** Network topological properties of the combined interaction network

Number of nodes	13,460
Number of edges	141,296
Mean degree	21
Mean clustering coefficient	0.17
Mean shortest path	3.6
Max diameter	12

#### Benchmark of drug-disease associations

All the known associations between chemicals (or equivalently, drugs) and disorders or its descendants are got from Comparative Toxicogenomics Database (CTD) in May 2014 as our benchmark [29]. CTD contains two kinds of chemical–disease associations: curated and inferred. Curated associations are extracted from the published literature by CTD biocurators and inferred associations are established via CTD–curated chemical–gene interactions. In our study, we extract both curated and inferred associations, which can help researchers develop hypotheses about environmental diseases and their underlying mechanisms.

#### Functional enrichment analysis

In order to validate our method further, we utilize the Database for Annotation, Visualization and Integrated Discovery (DAVID) to perform functional enrichment analysis [33, 34] on the gene sets of predicted drug-disease pairs. With the genes as inputs, we observe the overlapping of enriched KEGG pathways between drugs and diseases. With Benjamin multiple testing correction method [35], the enrichment *p*-value is corrected to control family-wide false discovery rate under certain rate (e.g. ≥ 0.05).

### Compute distance between modules

The disease- or drug-associated genes interacting with each other suggests that they tend to cluster in the same neighborhood of the interactome and form a disease module or a drug module, a connected subgraph that contains all molecular mechanisms of a disease or a drug. Therefore, the accurate evaluation of relationships between disease modules and drug modules is a very important step to identify potential drug-disease associations. Because the interactome remains incomplete, Menche et al. [27] proposed a new definition of module distance in 2015. Here, it is named as Module Distance for convenience. Given two modules marked as A and B, the Module Distance between them is defined as *s*_AB_ [27]:

1 $$ {s}_{\mathrm{AB}}\equiv <{d}_{\mathrm{AB}}>-\frac{<{d}_{\mathrm{AA}}>+<{d}_{\mathrm{BB}}>}{2} $$

< *d*_AA_ > represents mean shortest distance between each node and all the other nodes within module A. < *d*_BB_ > represents mean shortest distance between each node and all the other nodes within module B. < *d*_AB_ > represents mean shortest distance between nodes within module A and nodes within module B.

**Fig. 1:**
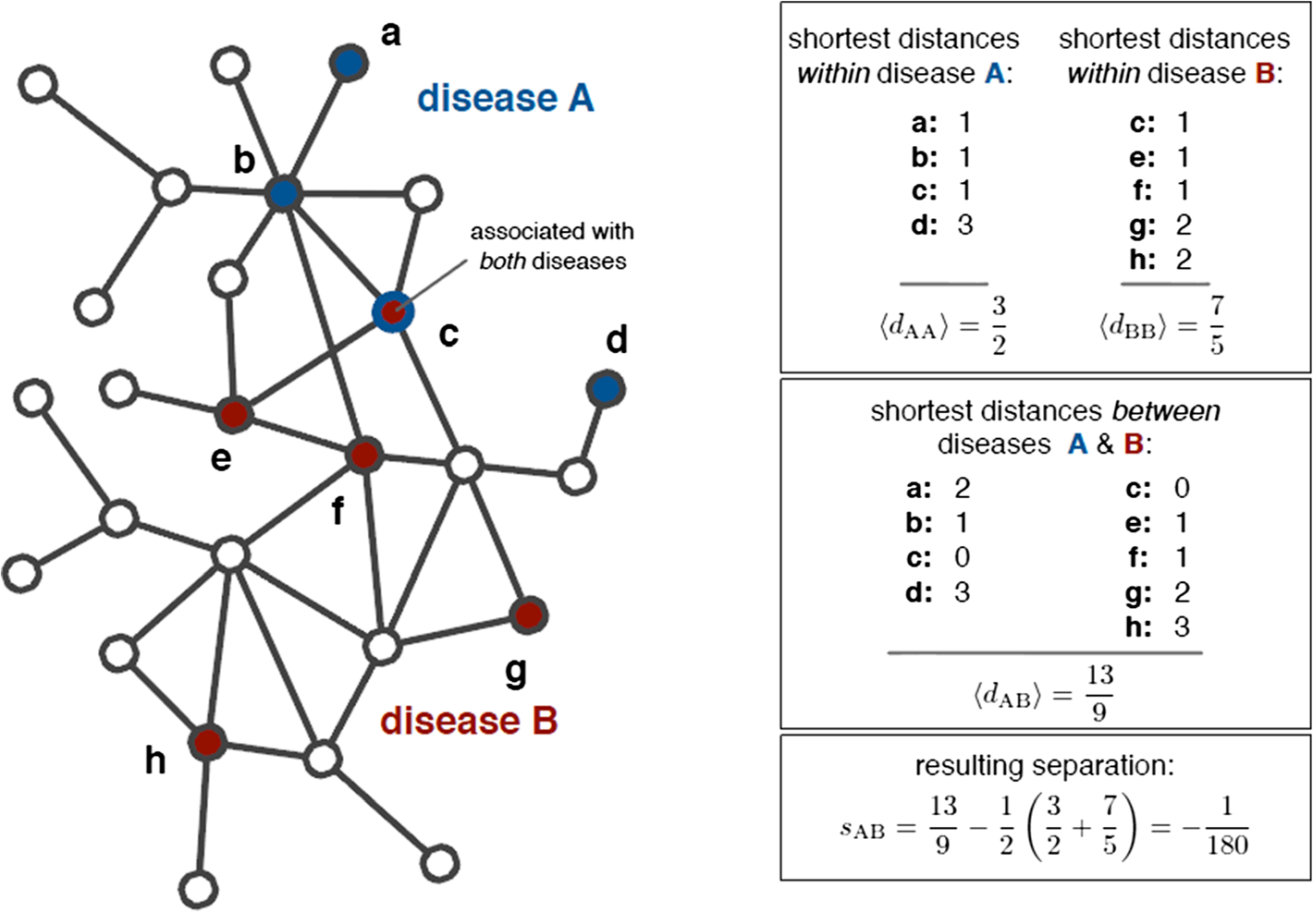
An example for calculating the distance between disease module A and B [27]. Blue and red nodes represent nodes belonging to module A and module B respectively. Node *c* is a shared node of modules A and B

A simple example for calculating the distance between two disease modules A and B is shown in Fig. 1 [27]. In Fig. 1, the four nodes within disease module A, {*a*, *b*, *c*, *d*}, are labeled by blue and the other five nodes within disease module B, {*c*, *e*, *f*, *g*, *h*}, are labeled by red. For node *a* in module A as an example, its shortest distances to *b*, *c* and *d* are 1, 2 and 5 respectively, so its shortest distance with all the other nodes within module A is 1. Similarly, the shortest distances of *b*, *c* and *d* in module A are 1, 1 and 3 respectively (see Fig. 1). Therefore, the mean shortest distance within module A, <*d*_AA_>, is (1 + 1 + 1 + 3)/4 = 2/3. In this way, the shortest distance in module B, <*d*_BB_>, is (1 + 1 + 1 + 2 + 2)/5 = 7/5. Then we calculate the mean shortest distance between modules A and B, <*d*_AB_>. Firstly, the shortest distances for all the node pairs between module A and module B are calculated. As shown in Fig. 1, node *a* in module A is closest to node *c* in module B, so the shortest distance between node *a* and module B is 2. In the same way, the mean shortest distance between modules A and B, <*d*_AB_>, can be got and shown in Fig. 1. Finally, according to formula (1), the distance between modules A and B, *s*_AB_, is calculated and its value is negative. The reason is that module A and module B share a common node *c*.

### Construct drug-disease associations based on Module Distance scores

Based on Module Distance, we calculate the distances between 55 cancer modules and 3,594 drug modules. First, all the genes related to drugs and diseases are mapped to the combined protein network. For each drug and each disease, their related genes form a drug module and a disease module respectively. Then, using the formula (1), we can calculate the distance between each drug-disease module pair. Finally, in order to make the distances score be proportional to the drug-disease correlations, we process the distance scores as follows. At the beginning, we turn all distances into positive by adding the minimum distance score to each distance, and then we get their reciprocals. At last, we use maximum-minimum to normalize all the distances. Consequently, the larger the distance score, the more related between drug and disease. Eventually, we obtain (55 × 3594) disease-drug associations. In order to obtain more meaningful results and filter possible false positive correlations, we will filter the distances by *p*-value in the following section.

### Filter drug-disease distances by *p*-value

**Fig. 2:**
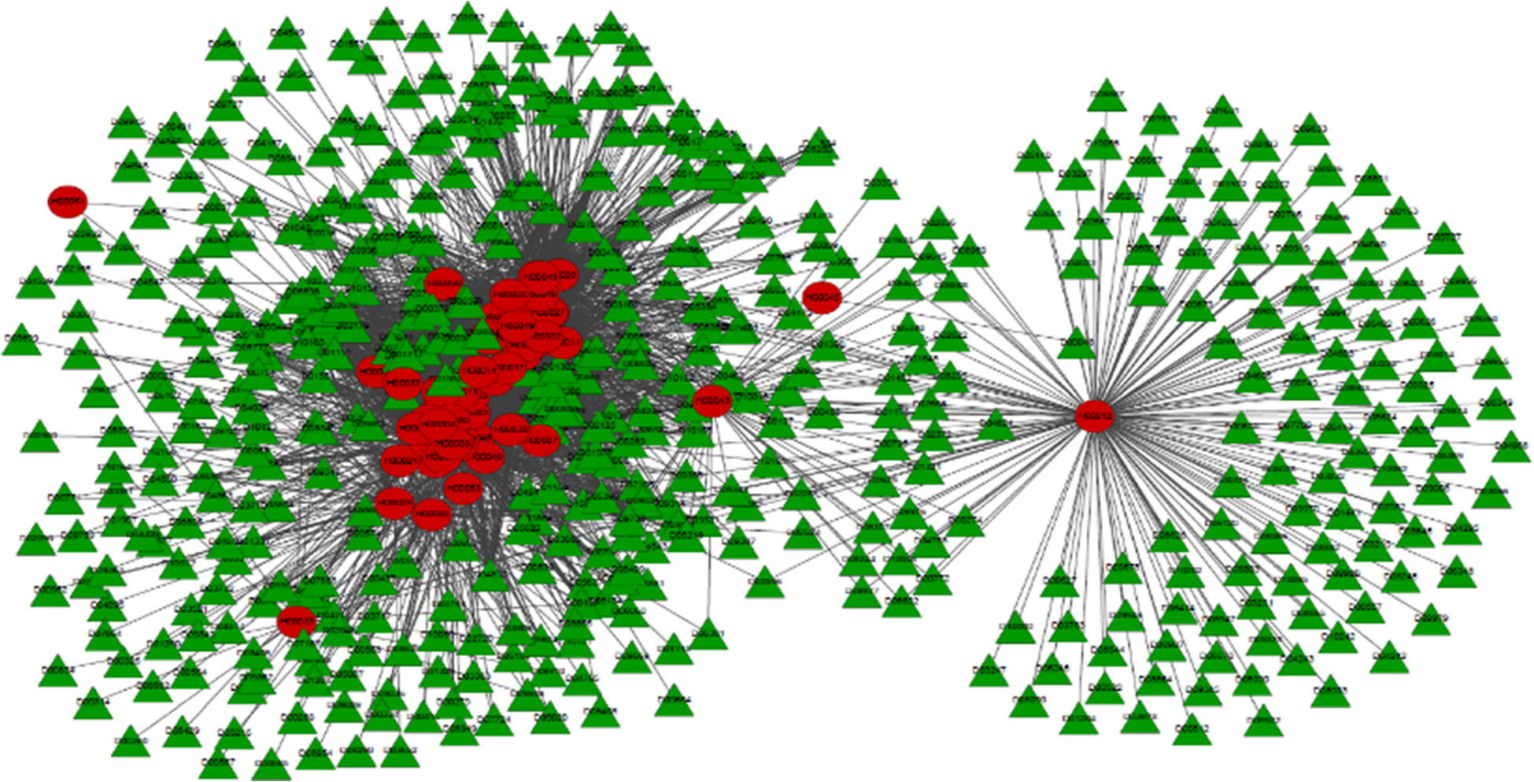
Disease-drug associations after filtering by *p*-value (*p*-value ≤ 0.01). Red circular and green triangle nodes represent diseases and drugs respectively

Based on the combined protein interaction network, we generate 10,000 random networks which keep the degrees of nodes in the original network. Then in each of the random networks, we calculate the distances between drug modules and disease modules by using Module Distance (see formula (1)). Finally, for each one in 55 × 3594 disease-drug associations, we can get its corresponding *p*-value. We discard all the edges whose *p*-values are not lower than 0.01. As a result, we obtain 3,027 drug-disease associations and they are presented in Fig. 2.

## Results and discussion

### CTD benchmark verification

We rank the 3,027 remained drug-disease associations in descending order on the basis of their scores. According to the definition of the distance between a drug-disease pair, the drug-disease pairs with higher scores are what we need. In order to analyze our results more targeted and find more valuable associations, we focus on the top-100 drug-disease associations for further analysis by CTD benchmark. Their scores are more than 0.67.

**Fig. 3:**
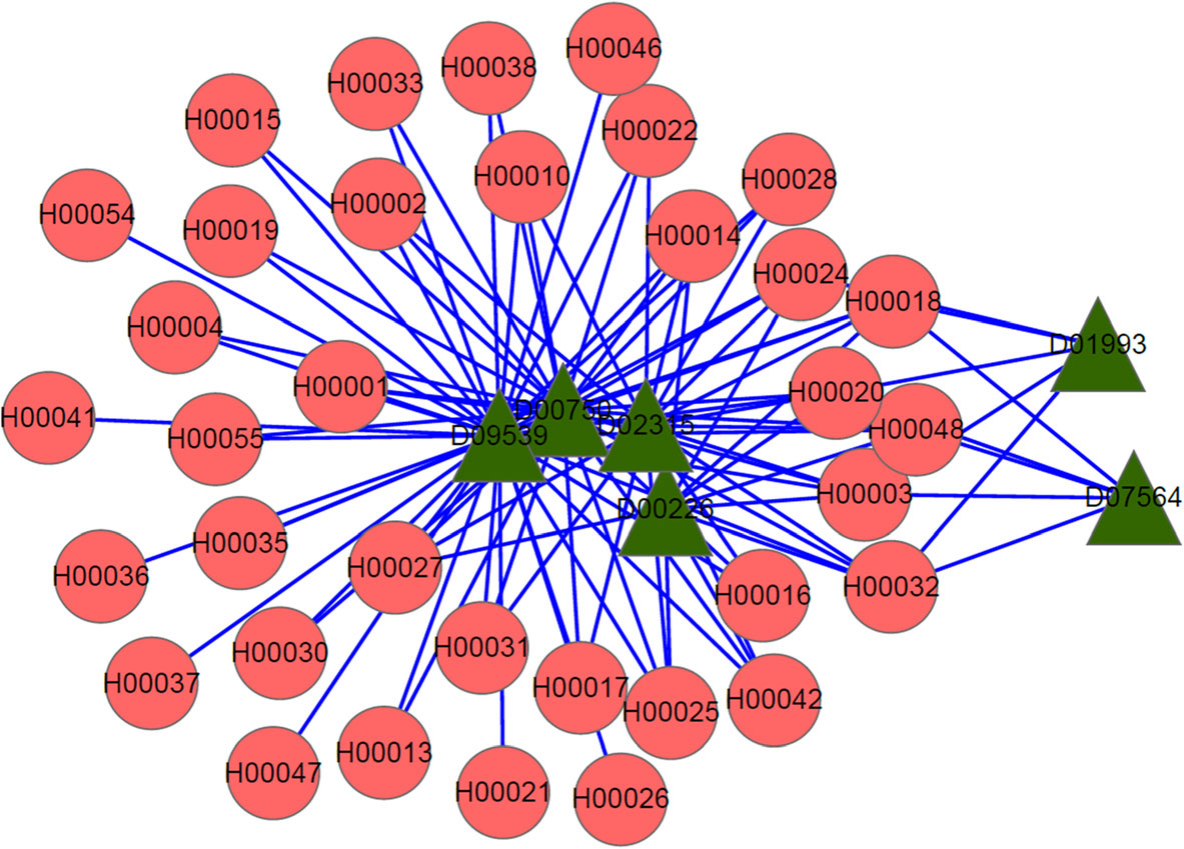
The top-100 predicted drug-disease relationships. The green triangle nodes represent drugs and the red circular nodes represent diseases

For the top-100 drug-disease relationships, they relate to 6 drugs and 35 diseases in all. Their connected network shown in Fig. 3 is a drug-disease bipartite graph with 100 links between 6 drugs and 35 diseases. The green triangle nodes represent drugs and the red circle nodes represent diseases. From Fig. 3, we find D09539 (drug name: Gabapentin enacarbil), D00750 (drug name: Levamisole hydrochloride) and D02315 (drug name: Oleic acid), are associated with 35, 27 and 18 diseases respectively. The other three drugs, D00226 (drug name: Amifostine), D01993(drug name: Polidocanol), and D07564 (drug name: Allopurinol), are associated with the remained 20 associations. Table 2 gives the summary information of the six drugs based on CTD, including the number of existing diseases (represented by Ne), the number of predicted diseases (represented by Np) and the percentage, i.e. Ne/(Ne + Np).

**Table 2:** The summary information of D09539, D00750 and D02315 based on CTD

KEGG DrugID	The number of existing diseases (Ne)	The number of predicted diseases (Np)	The percentage (Ne/(Ne + Np)
D09539	26	9	74.3%
D00750	18	9	66.7%
D02315	12	6	66.7%
D00226	10	0	100%
D07564	5	0	100%
D01993	0	5	0

In Table 2, we can find in the top-100 results, the 10 associations related to D00226 and 5 ones related to D07564 are all found in CTD database, i.e. their percentages are 100%. In a certain degree, the exciting results show the reliability of our algorithm. For D01993, it only relates to three diseases in CTD database: “Dermatitis, Allergic Contact”, “Facial Dermatoses” and “Hand Dermatoses”, so it is hard to find its existing diseases. The reason may be the interactome and the drug gene list remain incomplete and biased toward much-studied drugs genes and drug mechanisms. Furthermore, for D09539, D00750 and D02315, there is a total of 80 associations in the top 100 relationships related to them. Therefore, in the following sections, we will make a further analysis on D09539, D00750 and D02315 and their related diseases one by one.

**Fig. 4:**
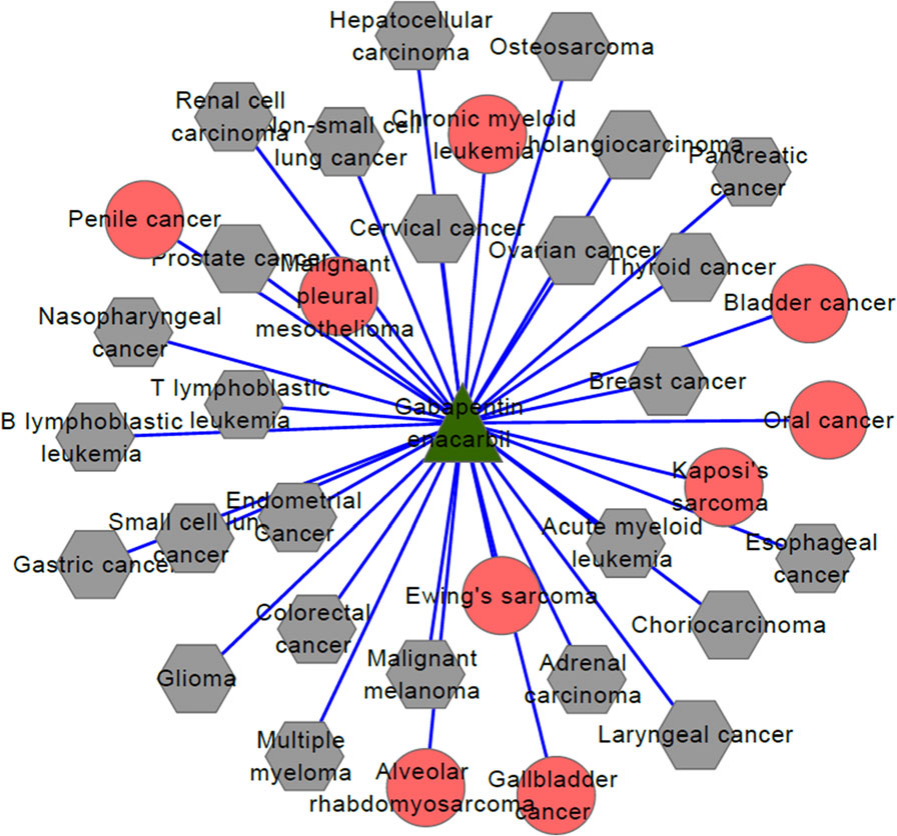
D09539 (drug name: Gabapentin enacarbil) and its related diseases. Green triangle node represents drug D09539, gray hexagonal nodes represent known related diseases in CTD and red circular nodes represent new predicted related diseases

**Fig. 5:**
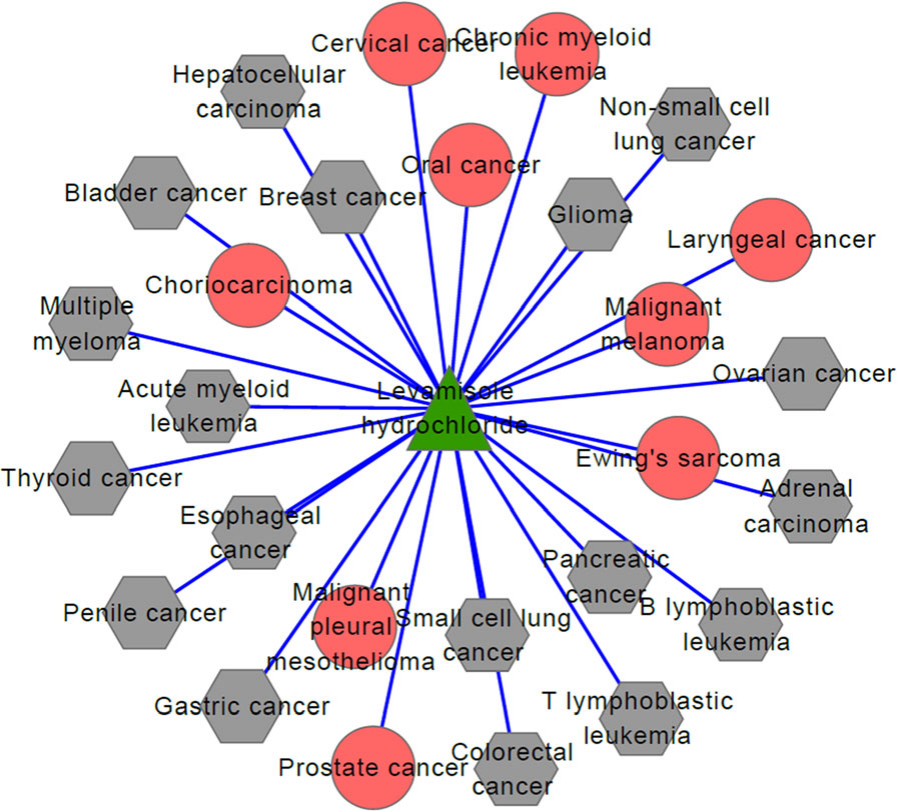
D00750 (Levamisole hydrochloride) and its related diseases. Green triangle node represents drug D00750, gray hexagonal nodes represent known related diseases in CTD and red circular nodes represent new predicted related diseases

**Fig. 6:**
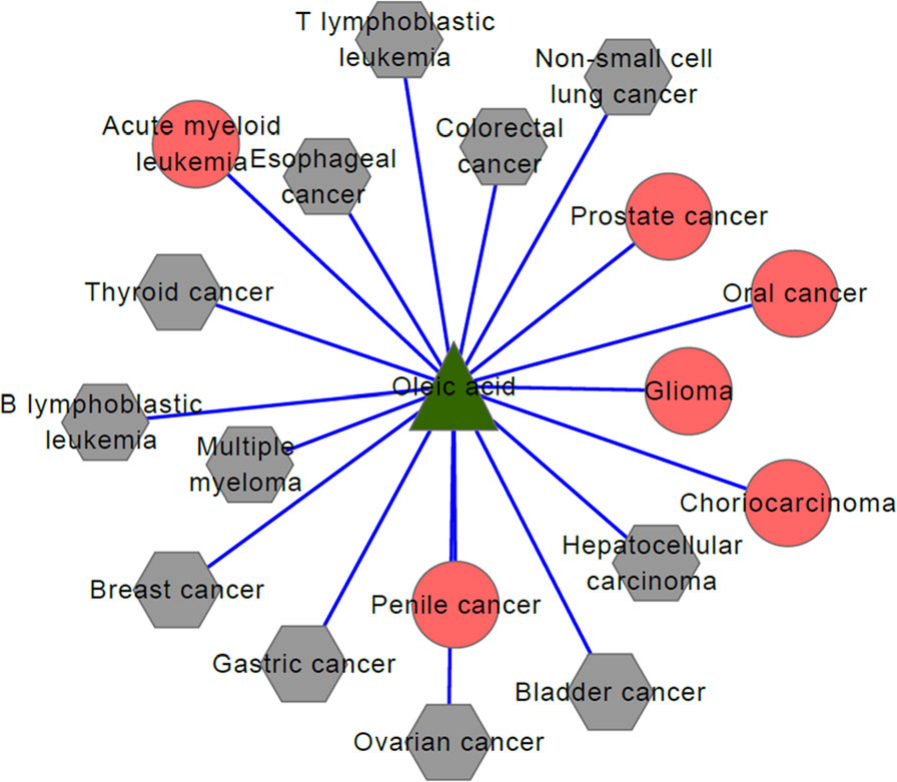
D02315(Oleic acid) and related disease network. Green triangle node represents drug D02315, gray hexagonal nodes represent known related diseases in CTD and red circular nodes represent new predicted related diseases

For the first drug D09539 (drug name: Gabapentin enacarbil), its connections with related diseases are shown in Fig. 4. In the following figures, Figs. 4, 5 and 6, green triangle nodes represent drugs, gray hexagonal nodes represent existing diseases in CTD and red circular nodes represent predicted related diseases. There are 35 diseases connected to D09539 (Gabapentin enacarbil) and 26 of them are recorded in CTD database. The percentage reaches up to 74.3%. Therefore, the remaining 9 diseases are likely to be related to D09539 (Gabapentin enacarbil). They may be new indications of Gabapentin enacarbil or its side effects.

The second drug D00750 (drug name: Levamisole hydrochloride) is connected to 27 diseases and their connections are shown in Fig. 5. By verifying in CTD database, we find 18 of 27 diseases are known associations with Levamisole hydrochloride and only 9 diseases are newly predicted results. The prediction accuracy is more than 50%, i.e. 66.7%. We estimate that Levamisole hydrochloride may treat some of the nine predicted diseases or cause some of them.

Figure 6 shows the associations of the third drug D02315 (drug name: Oleic acid) and its related disease. In the same way, we use CTD benchmark to analyze our results. We find 18 diseases are related to Oleic acid: 6 of them are predicted ones and the other 12 diseases have been recorded in CTD database. The percentage also reaches up to 66.7%. No matter what kind of relationship between Oleic acid and the six new diseases, the results are helpful in drug discovery and disease treatment.

Through analyzing our results based on CTD benchmark, we find the prediction accuracies of three drugs (D09539, D00750 and D02315) are all relatively high, more than 50%. On the other hand, the facts indicate that those diseases having no records in CTD are likely to be the new indications of drugs. Therefore, in the following section, we will use KEGG functional enrichment analysis and literature mining to further verify the reliability of our predicted potential associations.

### KEGG pathway functional enrichment analysis and literature verification

In the above section, the top-100 results are validated by CTD benchmark. We mainly analyze three drugs, whose associated diseases are 80% of the top-100 results. After our analysis, we obtain 9, 9 and 6 potential diseases for D09539 (drug name: Gabapentin enacarbil), D00750 (drug name: Levamisole hydrochloride) and D02315 (drug name: Oleic acid) respectively. Their details are shown in Table 3. We perform KEGG pathway enrichment analysis on the target sets of drugs and their related diseases with the functional annotation tool of DAVID [33, 34]. If a drug has overlapped KEGG pathways with a disease, the drug and the disease may have great relevance. The drug can probably treat or cause the disease through acting on the overlapping pathways. For DAVID, EASE Score, a modified Fisher Exact *P*-Value, is used as a threshold for gene-enrichment analysis [35]. It ranges from 0 to 1. When Fisher Exact *P*-Value is 0, it represents perfect enrichment. We set it as 0.01.

**Table 3:** Three drugs, their corresponding targets and related diseases

Drugs	Gabapentin enacarbil (D09539)	Levamisole hydrochloride (D00750)	Oleic acid (D02315)
Targets of Drug	55799; 781; 9254; 93589	1136; 251	51228; 5375
Related Diseases	**Penile cancer;** **Choriocarcinoma;** Malignant pleural mesothelioma; **Oral cancer;** Ewing's sarcoma; Chronic myeloid leukemia; Alveolar rhabdomyosarcoma; **Kaposi's sarcoma;** **Gallbladder cancer**	Prostate cancer; Choriocarcinoma; Ewing's sarcoma; Oral cancer; Malignant pleural mesothelioma; Laryngeal cancer; Chronic myeloid leukemia; Malignant melanoma; Cervical cancer	Prostate cancer; Acute myeloid leukemia; Glioma; Penile cancer; Choriocarcinoma; Oral cancer

Boldface diseases represent they have overlapped KEGG pathways with drugs

Gabapentin enacarbil (KEGG DrugID: D09539) is a prodrug for the anticonvulsant and analgesic drug gabapentin [36]. It is used for treating restless leg syndrome (RLS) and postherpetic neuralgia (PHN) [37, 38]. Although the exact mechanism of action of gabapentin in RLS and PHN is unknown, it is presumed to involve the descending noradrenergic system, resulting in the activation of spinal alpha2-adrenergic receptors. There are five caners, H00025 (Penile cancer), H00028 (Choriocarcinoma), H00016 (Oral cancer), H00041 (Kaposi's sarcoma) and H00047 (Gallbladder cancer), have overlapped KEGG pathways with Gabapentin enacarbil (shown in Table 3 marked as boldface). "MAPK signaling pathway" is their overlapped pathway (shown in Table 4 marked as boldface), which has been found related to multiple human diseases, including cancer [39]. In fact, Gabapentin enacarbil was denied approval by the U.S. Food and Drug Administration (FDA) in February 2010, citing concerns about possible increased cancer risk shown by some animal studies. KEGG enrichment analysis shows that four caners still have no overlapping with Gabapentin enacarbil (D09539) and also have not found relationships through literature mining. The reason is possible that the studies on these four diseases are still very limited.

**Table 4:** Gabapentin enacarbil and its related KEGG pathways

Drug	D09539(Gabapentin enacarbil)
Pathways Related to Drug	Arrhythmogenic right ventricular cardiomyopathy (ARVC) Cardiac muscle contraction Hypertrophic cardiomyopathy (HCM) Dilated cardiomyopathy **MAPK signaling pathway**

Boldface pathway represents overlapped one

For the remaining two drugs Levamisole hydrochloride (D00750) and Oleic acid (D02315), they have no overlapped KEGG pathways with their related diseases because the two drugs have no related KEGG pathways. Levamisole is a drug used to treat parasitic worm infections [40]. It has also been studied as a method to stimulate the immune system as part of the treatment of cancer [41]. Its nine related diseases are all cancers. Furthermore, studies demonstrate that the role of levamisole immunotherapy is as an adjuvant to radiotherapy in Oral cancer [42, 43]. For Malignant melanoma, the degree of improvement experienced by the patients that were treated by levamisole is of sufficient magnitude to warrant further investigation of this dose of levamisole as adjuvant treatment in patients with melanoma [44]. The results of Pulay and Csömör [45] and reference to pertinent literature indicate the possible effects of levamisole are discussed, as well as possibilities and place of the drug in the therapy of cervical cancer.

The last drug Oleic acid is a common monounsaturated fat that occurs naturally in various animal and vegetable fats and oils. Monounsaturated fat has been related to decreased low-density lipoprotein (LDL) cholesterol [46], so Oleic acid may be effective for the hypotensive (blood pressure reducing) [47]. Shannon et al. [48] found Monounsaturated fatty acids and the alpha-linolenic:eicosapentaenoic ratio were associated with reduced risk of prostate cancer. However, oleic and monounsaturated fatty acid levels in the membranes of red blood cells are associated with increased risk of breast cancer [49], although the consumption of oleate in olive oil is associated with a decreased risk of breast cancer [50].

## Conclusions

Because of the incompleteness of protein interactomes and the limited knowledge of disease genes and drug genes, we propose a new method based on a distance between two modules to predict drug-disease association. The distance is named Module Distance for convenience, which is originally defined to solve the incompleteness of human interactome. First, we project disease genes and drug genes to a combined protein interaction network respectively. Then based on Module Distance, we calculate the distances between drug genes and disease genes, and make a further processing to the distances before being the relationships of drug-disease pairs. Also, we filter possible false positive drug-disease correlations by *p*-value. Finally, we validate the top 100 associations related to six drugs by CTD benchmark. Three main drugs are further analyzed by KEGG pathway enrichment and literature mining, because they are related to 80 associations. The experimental results are encouraging. Both the positive and negative associations can be predicted. Our study offers opportunities for future toxicogenomics and drug-disease discovery.

## Abbreviations

BiRW: Bi-Random walk
CTD: Comparative Toxicogenomics Database
DAVID: the Database for Annotation, Visualization and Integrated Discovery
FDA: Food and Drug Administration
KEGG: Kyoto Encyclopedia of Genes and Genomes
LDL: Low-Density Lipoprotein
NSCLC: Non-Small Cell Lung Cancer
PHN: PostHerpetic Neuralgia
PREDICT: PREdicting Drug IndiCaTions
RLS: Restless Leg Syndrome
WES: Weighted Ensemble Similarity
